# Genetic Determinants in *HLA* and Cytochrome P450 Genes in the Risk of Aromatic Antiepileptic-Induced Severe Cutaneous Adverse Reactions

**DOI:** 10.3390/jpm11050383

**Published:** 2021-05-07

**Authors:** Ali Fadhel Ahmed, Chonlaphat Sukasem, Majeed Arsheed Sabbah, Nur Fadhlina Musa, Dzul Azri Mohamed Noor, Nur Aizati Athirah Daud

**Affiliations:** 1 Discipline of Clinical Pharmacy, School of Pharmaceutical Sciences, Universiti Sains Malaysia, Pulau Pinang 11800, Malaysia or alifadhel1131990@gmail.com (A.F.A.); dzulazri@usm.my (D.A.M.N.); 2 Department of Pathology, Faculty of Medicine Ramathibodi Hospital, Mahidol University, Bangkok 10400, Thailand; chonlaphat.suk@mahidol.ac.th; 3 Laboratory for Pharmacogenomics, Somdech Phra Debaratana Medical Center (SDMC), Ramathibodi Hospital, Bangkok 10400, Thailand; 4 The Thai Severe Cutaneous Adverse Drug Reaction (THAI-SCAR) Research Group, Chulalongkorn University, Bangkok 10330, Thailand; 5 Advanced Research and Development Laboratory, Bumrungrad International Hospital, Bangkok 10110, Thailand; 6 Forensic DNA for Research and Training Centre, Alnahrain University, Baghdad 64074, Iraq; majeedbio@gmail.com; 7 Human Genome Center, School of Medical Sciences, Universiti Sains Malaysia, Kubang Kerian, Kota Bharu 16150, Malaysia; fadhlina@usm.my

**Keywords:** HLA, cutaneous adverse drug reaction, SCAR, genetic polymorphism, antiepileptics, CYP450 enzymes

## Abstract

Adverse drug reaction (ADR) is a pressing health problem, and one of the main reasons for treatment failure with antiepileptic drugs. This has become apparent in the event of severe cutaneous adverse reactions (SCARs), which can be life-threatening. In this review, four hypotheses were identified to describe how the immune system is triggered in the development of SCARs, which predominantly involve the human leukocyte antigen (HLA) proteins. Several genetic variations in HLA genes have been shown to be strongly associated with the susceptibility to developing SCARs when prescribed carbamazepine or phenytoin. These genetic variations were also shown to be prevalent in certain populations. Apart from the HLA genes, other genes proposed to affect the risk of SCARs are genes encoding for CYP450 drug-metabolising enzymes, which are involved in the pharmacokinetics of offending drugs. Genetic variants in CYP2C9 and CYPC19 enzymes were also suggested to modulate the risk of SCARs in some populations. This review summarizes the literature on the manifestation and aetiology of antiepileptic-induced SCARs, updates on pharmacogenetic markers associated with this reaction and the implementation of pre-emptive testing as a preventive strategy for SCARs.

## 1. Introduction

An adverse drug reaction (ADR) is defined as a noxious or unintended response of the body to regular exposure to chemical materials such as drugs, for the purpose of prophylaxis, diagnosis or therapy of a disease, or modification of physiological functions of organs [1]. ADRs are common at the community level and account for about 6.5% of all hospitalized cases, and incur increased attention, health care and community costs in 15% of patients. Statistics in the United States of America indicate that ADRs are responsible for more than 100,000 deaths annually, making ADR the sixth leading cause of death in the States, with similar statistics seen in the United Kingdom [2,3].

Several antiepileptic drugs (AEDs) are commonly associated with adverse skin reactions [4]. Severe and life-threatening skin reactions are referred to as severe cutaneous adverse reactions (SCARs) [5]. SCARs which occur following the use of certain drugs are a type of delayed hypersensitivity reaction that is idiosyncratic, unpredictable and dose-independent. SCARs account for 15–20% of all adverse drug reactions, which include Stevens-Johnson syndrome (SJS), toxic epidermal necrolysis (TEN) and drug reaction with eosinophilia and systemic symptoms (DRESS). Statistics indicate that the annual incidence of SJS is 1.2–6.0 per million, while the incidence of TEN is 0.4–1.2 per million annually [6,7]. Both reactions are characterized by high mortality and morbidity rates despite the low incidence, as the mortality rate for SJS is 1–5% while for TEN it is 25–30% [8,9]. SCARs are most commonly observed among patients taking carbamazepine (CBZ), phenytoin (PHT) and lamotrigine, and typically occur within the first three months of initiation [8].

Knowledge of patients’ genetic information might help in preventing antiepileptic-induced SCARs. Several genetic variations, especially in the genes encoding for human leukocyte antigen HLA-A, HLA-B and cytochrome P450 enzymes have been significantly associated with a higher risk of developing a SCAR [9,10,11]. Identifying the type of SCAR is also essential for determining the HLA alleles and the P450 isoforms associated with this hypersensitivity syndrome. The findings of this association will shed light on the predisposing genetic risk factors in SCARs and provide a means for preventive measures, such as a pre-emptive screening of HLA and CYP450 markers before the initiation of drug treatment [12]. This is in line with the emerging approach of precision medicine where prescribed medication takes individual genetic variability into consideration. This review further focuses on the genetic variations of several genes involved in the mechanism of antiepileptic-induced SCARs, and the potential contribution of pre-emptive testing as part of the genotype-guided therapeutics for the prevention of SCARs.

## 2. Severe Cutaneous Adverse Drug Reactions (SCARs) and AEDs Use

SCARs linked with the use of medications are a type of delayed hypersensitivity reaction that is idiosyncratic, non-predictable and independent of dose. SCARs pose a challenge in the clinical management of patients, as they are related to increased mortality and morbidity, long-term subsequent medical events and high healthcare costs [13].

Skin rash is a common manifestation of ADR in affected individuals. SCARs constitute a large variety of clinical phenotypes, from maculopapular exanthema (MPE) to hypersensitivity syndrome (HSS), SJS, TEN, DRESS and acute generalized acute exanthematous pustulosis (AGEP) [14]. A milder form of cutaneous adverse reaction is MPE, a rash that is mild, self-limited and usually resolved after the offending drugs are withdrawn. Unlike MPE, SCARs have serious morbidity, involve systemic manifestation and pose high mortality [15].

About 3% of patients on antiepileptic drugs may develop cutaneous eruptions [16]. A mild form of MPE may occur in about 2.8% of AED users, with phenytoin, lamotrigine and carbamazepine displaying the highest rates (5.9%, 4.8% and 3.7%, respectively), and these reactions may resolve naturally within 3–20 days [17]. Meanwhile, more severe types of cutaneous ADRs including HSS, SJS, TEN and DRESS are less common [18,19]. The histopathology of MPE, DRESS and SJS/TEN is predominated with exocytosis and lymphocyte and macrophage infiltration. In severe cases of SJS/TEN, extensive keratinocyte necrosis is observed. The severity of inflammatory infiltrate and epidermal manifestation increases from MPE to DRESS and SJS/TEN [18].

## 3. Clinical Manifestations of SCARs

**Figure 1:**
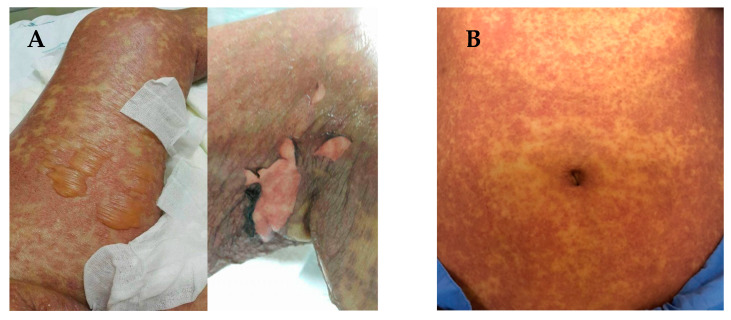
(**A**) Manifestations of Stevens-Johnson syndrome (SJS) and toxic epidermal necrolysis (TEN), in which the skin begins to blister and peel. Erythroderma, extensive skin lesions, aggressive detachment of the epidermis and erosion of mucous membranes can be observed; (**B**) dermatologic manifestations of drug reaction with eosinophilia and systemic symptoms (DRESS) typically consist of diffuse pruritic macular and urticarial rash. Facial oedema and periorbital areas with scale and crust around the nose and lip can be found. All patients have provided informed consent before being enrolled and provided the pictures.

SJS and TEN are characterized by sloughing of the skin, mucosal membranes and the surface of the eye via immune mechanisms that lead to cell death or necrosis [19]. The early stages of SJS/TEN clinical manifestation include fever, malaise, flu-like symptoms, and also symptoms involving eyes, ear, nose and throat for a few days or up to 2 weeks prior to cutaneous manifestations. Cutaneous manifestations of SJS begin with skin pain or a burning sensation (Figure 1A,B). It first appears on the face, pre-sternal area of the upper trunk and extremities, and involves erythroderma, purpura, pustules and swelling of the affected area [20]. Symptoms are typically manifested in the proximal parts of the extremities, while the distal parts are relatively spared [21]. In a short period of time, erythematous macules or diffuse erythema will develop over the trunk and extremities. As the red areas develop, the central dusty necrotic areas expand with the subsequent growth of bullae. As the disease progresses, the layers of the full-thickness epidermis will separate, exposing dark red, moist dermis resembling extreme second-degree burns [22,23].

A positive Nikolsky sign is significant diagnostic evidence and precedes the occurrence of life-threatening events [24,25]. There is less than 10% and over 30% epidermal detachment in SJS and TEN, respectively. Any disorder with between 10% and 29% epidermal detachment is typically diagnosed as overlapping with SJS/TEN [26]. The clinical presentation of these reactions may also differ among individuals. In one confirmed case of SJS, only mucosal membrane lesions without skin lesions were present in a 14 year-old child [27]. Mucosal membrane involvement may occur in 85% to 95% of SJS and TEN patients; while involvement of conjunctivae, mucous membranes of the nares, mouth, oropharynx, anorectal junction, vulvovaginal area and urethral meatus may occur in 85% to 95% of SJS and TEN patients [28].

Early diagnosis of SCARs that helps in the identification of the culprit drugs is important in the acute stages of the reaction. A prompt recognition helps to improve the management of the disease and limits long-term sequelae. Classification of SCARs is also important in identifying causal drugs. Studies have shown that the interval between drug intake and SCARs onset differ according to the type of SCARs. SJS/TEN has a shorter latency period compared to DRESS [29]. Assessment scores and tools developed to assist in investigation of SCARs to determine clinical patterns and identify causal drugs are discussed in the following section.

## 4. Phenotyping and Causality of SCARs

The clinical phenotype of SCARs is defined by the disease characteristics (i.e., phenotypic traits) that explain discrepancies between persons with the reactions in terms of clinically significant outcomes (such as symptoms, extent and severity of rash, involvement of other organs, as well as laboratory and clinical parameters) [29,30]. Clinical phenotyping of SCARs was proposed by RegiSCAR (European Registry of Severe Cutaneous Adverse Reactions), a multinational collaborative research consortium previously known as EuroSCAR [31]. The RegiSCAR group operates as a registry of clinical data and biological samples, and provides continuous surveillance on new drugs and guidelines for SCARs. The aim of this group is to promote the safe use of drugs while reducing the medical and economic burden of SCARs on public health. To prevent misdiagnosis, RegiSCAR defined consensus diagnosis criteria for each SCAR phenotype. SCAR cases were investigated using standardized questionnaires to obtain detailed information on clinical phenotypes, including collecting clinical photographs and skin biopsies and associated medical conditions. Other phenotypes like type of skin rash, hospitalization and organ involvement are also included in the assessment criteria in the diagnosis of HSS/DRESS [29].

In the determination drug causality, the assessment criteria include non-genetic factors such as drug exposure, rechallenge, disease aetiology and previous report of similar cases [31]. The RegiSCAR group reported that the causality of SCARs could be attributed to other high-risk medications and that misdiagnoses were frequent. Overlapping SCAR phenotypes were rare, as SJS and TEN are known to be variants of the same disease, while SJS/TEN and DRESS are two distinct diseases [29,32].

Several algorithms were used to perform the causality assessment of SCARs objectively, as a standardized approach is important to establish an accurate and reproducible diagnosis. The algorithms used for drug causality assessment in cutaneous ADRs include the Naranjo algorithm, the French pharmacovigilance causality score test and the RUCAM algorithm [31,33]. From the RegiSCAR findings, a specific algorithm for drug causality was constructed, known as the drug causality algorithm for epidermal necrolysis (ALDEN), which has gained prominence as it is frequently applied in recent SJS and TEN cases [31].

Assessing drug causality is also a challenge due to incomplete reporting of drug exposure. Generally, for SCARs assessment, it relies mainly on cutaneous manifestation and clinical presentation, duration of the eruptions, associated symptoms and latency time between starting the drug and eruption onset. The distribution and physical examination of the skin lesions coupled with skin biopsy for histological testing are important for the accurate and quick diagnosis of SJS/TEN, as a diagnosis of SJS/TEN within 7 days of onset is associated with improved survival [18]. Using the study data, several parameters were considered in the estimation of drug risk, prognosis indexes, disease outcome and effects on treatments for SCARs. Other ongoing investigations include phenotype determination, lymphocytes’ antigenic specificity and susceptibility genes and associated single-nucleotide polymorphisms (SNPs) [34].

Not all individuals exposed to the offending drugs are affected, which might indicate a genetic predisposition towards this effect. Progress in the field of genetics has broadened our knowledge and ability to prevent ADR events by identifying the genetic markers responsible for such reactions among the affected patients [35]. Genetic markers found to be linked to an increased risk of SCARs are genes encoding for human leukocyte antigen (HLA), drug transporter proteins (e.g., ABCB1, SLCO1B1), drug-metabolizing enzymes (cytochrome P450), glucose-6-phosphate dehydrogenase (G6PD) and nucleoside diphosphate linked moiety X-type motif 15 (NUDT15) [36]. The following sections focus on genetic markers in HLA genes and genes encoding for cytochrome P450 enzymes.

## 5. Human Leukocyte Antigen (HLA) and Its Role in ADRs

The major histocompatibility complex (MHC) is a group of proteins on the cell surface that mediate immunological reaction to foreign molecules or antigens entering the body [37]. The MHC binds to foreign molecules and displays them on the surface of cells for recognition by corresponding T cells, followed by the release of immunological mediators as a response. Human MHC, also known as human leukocyte antigen (HLA) complex, is encoded by over 200 genes located on the short arm of chromosome 6 (6p21.3) [38]. HLA complex is divided into three major classes: class I, type II and class III. Class I consists of three genes: HLA-A, HLA-B and HLA-C. The HLA class I molecules are expressed on the membranes of most karyocytes and can provide CD8+ T cell endogenous peptides, the cytotoxic T cells. Meanwhile, class II MHC consists of six primary genes: HLA-DPA1, HLA-DPB1, H1LA-DQA1, HLADQB1, HLA-DRA and HLA-DRB1. The HLA class II is only expressed on immune cell surfaces such as exogenous peptides to CD4+ T-helper cells and dendritic cells. Occasionally, cross-presentation may occur, such as in viral infections [39].

All cells responsible for presenting peptides to immune cells express HLA class I in their surfaces. In general, old proteins are broken down from cells by a continuous process of collecting peptides to make new ones. Some of the broken peptide fragments bind with MHC molecules and are recognized by the body’s immune cells as “self-molecules”. If the broken cell has a pathogen, the pathogen peptides that bind to the molecules of the MHC are known as foreign (nonself) peptides and activate an immune response against the disease-causing antigens [40].

HLA genes are highly polymorphic, and the proteins bind to multiple types of peptides to be recognized as either “self-antigens” or “foreign antigens.” Genetic differences in HLA genes play a crucial role in determining the susceptibility of an individual to autoimmune diseases and other infections. It also plays a vital role in the success of organ transplants, as genetic compatibility of the HLA genes between the donor and the recipient is very important [34]. Several recent studies have also shown a significant correlation between HLA proteins and the risk of idiosyncratic ADRs [41,42]. The following sections discuss the proposed immune response related to SCARs and the genetic polymorphisms of several genes linked to the risk of SCAR caused by AEDs.

## 6. The Hypothesis of Immune Response in SCARs

ADRs are divided into type A, which is predictable, and type B, which is idiosyncratic [43,44,45]. Type A ADRs are directly caused by the pharmacology of drugs and commonly occur in a dose-dependent relationship; type B ADRs are not related to the pharmacology or dosage of drugs [44,45,46]. Idiosyncratic ADRs can be immune-mediated or non-immune-mediated [41]. Immune-mediated ADRs are rarely noticed during clinical trials and are difficult to predict. There are some rare cases where the drug induces an immune response by interacting with MHC molecules, but not much is known about the exact mechanisms associated with such ADRs [41,47].

**Figure 2:**
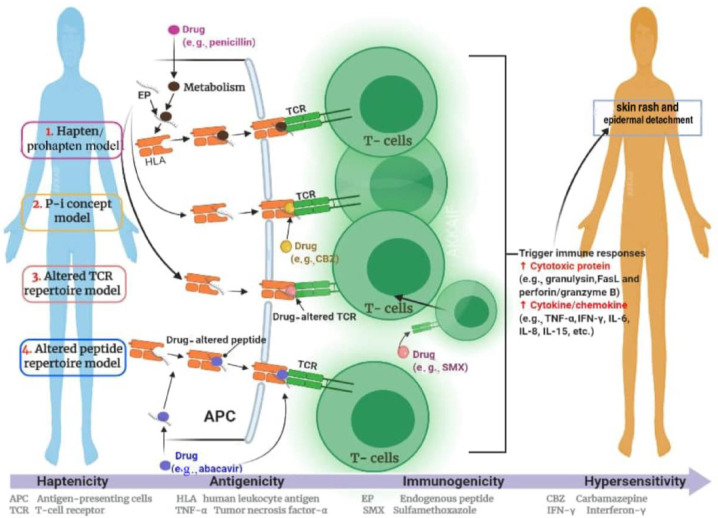
The hypothesis pertaining to the immune response mechanism during severe cutaneous adverse reaction.

Researchers developed four hypotheses to understand how the immune system is activated in an HLA-molecule-dependent manner in the development of SCARs: (i) the “hapten/prohapten” theory, (ii) the “p-i” concept, (iii) the “altered peptide repertoire” model and (iv) the “altered T cell receptor (TCR) repertoire” model [48,49]. Figure 2 illustrates the proposed mechanism of immune response in SCARs.

The first hypothesis, “hapten/prohapten”, suggests that a drug or its reactive compound can bind covalently to an endogenous peptide to form an antigenic hapten–carrier complex. The concept of this model is to establish covalent bonds between the medication or its reactive metabolites, self-peptides and HLA molecules. This is accompanied by the activation of drug-specific immune responses [50]. The second concept of pharmacological interaction with immune receptors, the “p-i” concept, assumes that the drug or its reactive metabolites may be directly, inversely or disproportionately linked to HLA and/or TCR without binding to the antigen peptide. This suggests that there is a pharmacological interaction with immune receptors, which implies stimulation of the immune system by noncovalent binding of a drug to T-cell receptors for antigens (p-i TCR) or human leukocyte antigens (p-i HLA). The consequences of these interactions are heterogeneous; clinically, it can lead to T-cell-mediated reactions such as Stevens-Johnson syndrome/toxic epidermal necrolysis, drug rash with eosinophilia and systemic symptoms, acute generalized exanthematous pustulosis and maculopapular eruptions. If the drug binds to the TCR, it can become stimulatory, and additional interaction with HLA/peptide complexes is necessary for full stimulation. In the “p-i” model, it is assumed that the classic antigen-processing pathway in antigen-presenting cells may be bypassed. The third model, the “altered peptide repertoire” model, proposes that the drug is bound closely to “self-peptides” and is presented in the form of a peptide repertoire given to HLA and TCR [50]. The drug is not related directly to HLA in the “altered peptide repertoire” model. For example, the “altered TCR repertoire” explains that a drug (e.g., sulfamethoxazole) binds to a certain TCR, changes the TCR conformation and has the ability to produce an HLA–self-peptide complex that triggers an immune response [50]. In the model of “altered TCR repertoire”, TCR acts as the initial molecular drug interaction. With the link of an offending drug presented to the HLA molecule or TCR, the HLA–drug–TCR combination can stimulate the activation of cell signalling pathways and result in an expansion of cytotoxic T lymphocytes.

## 7. Genetic Polymorphisms of HLA Genes

The MHC region has been associated with more diseases than any other region in the human genome. The MHC region is densely clustered with genes that have a strong linkage disequilibrium, making it challenging to pinpoint the exact causative variant and the related function. Disease resistance has been proposed to drive the evolution in MHC variation. By taking amino acid residue 57 of HLADQB in type I diabetes as an example, although autoimmune diseases are demonstrated to be associated with changes in the groove residues, it is still not fully understood how the changes mediate autoimmune diseases [51]. In humans, the HLA complex region might be the most polymorphic region. The polymorphisms in this region are not widely spread out, but are concentrated in the region encoding the peptide-binding groove. In the case of HLA class I, α1 and α2 domains involve variable amino acid residues and are the determinants of antigenic specificities of molecules. Of all the HLA genes, HLA-B is known to be the most polymorphic, with more than 4000 alleles [48,49].

Aside from its association to diseases, the HLA allele polymorphisms have been studied extensively to draw inferences about human migration and genetic diversity among different populations. For example, HLA-B*15, the most polymorphic allele in HLA-B locus, has a non-symmetrical distribution in the Asian population, whereby the alleles in northeast Asia are more prevalent in the global population. In contrast, the alleles found in the Southeast Asian (SEA) population are more specific to the SEA region. For example, the most common allele in B*15 lineage, HLA- B*15:01, is more prevalent in northeast Asia, whereas HLA-B*15:02 is more prevalent in the south of Asia spreading southwards into SEA. The distribution of HLA-B*15:02 supports the human migratory route into SEA [52,53]. HLA-B*15:13 is more specific, and is observed within the Indonesian archipelago of SEA, while HLA-A*31:01 is present in more than 15% of people with Japanese, Native American, South Indian (e.g., Tamil Nadu) and Arabic ancestry, in up to 10% of people with Han Chinese, Korean, European, Latin American and other Indian ancestry, and in up to 5% of African-Americans [9,50,54].

## 8. HLA Alleles and SCARs Induced by AEDs

SCARs are unpredictable and not dose-dependent. Substantial progress for underlying SCAR mechanisms has been made with the discovery of the association between HLA alleles and SCARs. In this section, we summarize recent updates in the identification of genetic markers involved in SCARs or hypersensitivity reactions induced by aromatic AEDs). The reported findings are summarized and listed in Table 1, while the frequency of each allele is presented in Table 2.

### 8.1. Carbamazepine-Induced SCARs

Carbamazepine (CBZ) is a drug widely used to treat epilepsy, bipolar disorder and trigeminal neuralgia [64,92,93]. While usually well-tolerated, up to 10% of patients may have a cutaneous ADR [93]. The only effective intervention in this situation is withdrawal or prevention of the use of this medication [7]. The clinical symptoms of CBZ-induced SCARs are similar to anticonvulsant hypersensitivity syndrome with an immune-based aetiology and genetic predisposition [11].

Previous reports demonstrated that carriers of the HLA-B*15:02 allele were associated with approximately a 100-fold risk of developing SJS/TEN with the use of CBZ15 [15,63,94,95,96]. The first documentation of the strong association (OR > 1000) between HLA-B*15:02 and SJS induced by CBZ was among Han Chinese patients [51,59]. Other reports among Malaysians, Han Chinese and Thai patients indicated that patients with CBZ-induced SJS carried HLA-B*15:02 alleles, while some patients also carried the HLA-B*15:21 allele [62,97,98]. One study among Han Chinese identified that 100% (44/44) of patients using CBZ therapy and presented with SJS/TEN syndrome were carrying the HLA-B*15:02 allele, while only 3% (1/301) of the CBZ -tolerant patients were HLA-B*15:02-positive [71]. Meanwhile, several studies reported a relationship between HLA-B*15:02 and SJS/TEN resulting from the use of CBZ therapy in many populations, including Africans, Koreans, Malaysians, Japanese, Caucasians and Indians [39].

The association between HLA-B*15:02 and CBZ-induced SJS/TEN is both ethnicity- and phenotype-specific. This association was successfully recorded in Han Chinese patients, and in Asian populations including Malays, Thais and Indians [62,99,100]. However, it was not commonly observed amongst Japanese and Caucasian patients [63,96,97]. Inconsistency and variation in the allele frequency between population groups is frequently observed. For example, the allele frequency of HLA-B*15: 02 was high amongst several Asian populations (0.12–0.157 in Malays, 0.057–0.145 in Han Chinese and 0.085–0.275 in Thais) but lower in other populations such as Koreans (0.004), Europeans (0.01–0.02) and Japanese (0.002) [101]. For other types of cutaneous ADRs like eosinophilia and systemic symptoms (DRESS), there were no reports on their risk associated with HLA-B alleles with the use of CBZ [71,102,103].

The established association between HLA-B*15:02 and the risk of CBZ-induced SCARs warrants preventative measures to ensure the safe use of this medication. Based on previous reports on the risk of this allele in patients receiving CBZ, the FDA and European Medicines Agency included warnings on drug labels advising the need for genotyping among patients from certain areas of Asia before starting CBZ therapy [104]. A pre-emptive screening for HLA-B*15:02 was introduced in some countries, and its clinical utility was demonstrated recently by a prospective cohort study in Taiwan [105]. In this study, around 4800 patients were genotyped, and 7.7% were positive for HLA-B*15:02. The patients were prescribed an alternative therapy to CBZ, and no patients developed SJS in the study, contrary to the expected ten cases based on past incidences [106]. A study in Thailand also found that the genotyping for HLA-B*15:02 was cost-effective in preventing the occurrence of SCARs [107].

Several HLA alleles constitute the HLA-B75 serotype commonly found in Southeast Asian populations, for example, HLA-B*15:21, HLA-B*13:01 and HLA-B*15:21 [78]. HLA-A*31:01 alleles were significantly associated with CBZ-DRESS in Europeans (*p* < 0.001; OR 57.6, 95% CI 11.0–340) [59]. The pathogenesis of these CBZ hypersensitivity reactions requires further research, as it remains unclear whether HLA-B*15:02 represents the true causal allele or if it is in linkage disequilibrium with another causal variant.

### 8.2. Phenytoin-Induced SCAR

Phenytoin is a commonly prescribed AED that can cause cutaneous ADRs, ranging from MPE to life-threatening SCARs such as DRESS and SJS/TEN [103]. A previous study showed a confirmed association between HLA-B*15:02 and phenytoin-induced SJS/TEN in Malay patients (61.5% (8/13 cases) vs. 21.9% (7/32 controls), OR 5.71; *p* = 0.016) and showed a significant association between HLA-B*15:13 and both phenytoin-induced SJS/TEN (53.8% (7/13 cases), OR 11.28; *p* = 0.003) and phenytoin-induced DRESS (100% (3/3 cases), OR 59.00; *p* = 0.003) [71].

Several studies reported the association between HLA-B*15:02 and phenytoin-induced SJS/TEN in the Han Chinese population [69,100]. Furthermore, a study by Hung et. al. found a genetic risk with several other alleles, including HLA-B*15:02, HLA-B*13:01, Cw*08:01 and DRB1*16:02 in a similar population [59]. Cw*08:01 is within the haplotype of B*15:02, and therefore its association can be explained by linkage disequilibrium. On the other hand, despite a statistically significant association for HLA-B*13:01 and DRB1*16:02 alleles [59], the sample size was reported to be small. The association between B*15:02 allele and phenytoin-induced SCARs could not be replicated in some other studies [103,108], while only one study reported the association between HLA-B*56:02 and phenytoin-related SCARs, including DRESS and SJS/TEN [108]. In the European population, there was no association found between HLA-A*31:01 and phenytoin-induced cutaneous ADRs [9,109].

### 8.3. Lamotrigine-Induced SJS/TEN SCARs

Lamotrigine is a new-generation AED, and several alleles have been associated with lamotrigine-induced SCARs. In Thai patients, HLA-A*02:07 and HLA-B*15:02 were associated with an increased risk of lamotrigine-induced cutaneous ADRs, while associations were also found between HLA-A*33:03, HLA-B*15:02, HLA-B*44:03 and lamotrigine-induced MPE [9]. Meanwhile, there are contradictory findings on the association between HLA-B*15:02 and lamotrigine-induced SJS/TEN [59,69,72,100]. In the Han Chinese population, one study found an association between HLA-A*33:03 and HLA-B*15:02 and lamotrigine-induced MPE [110]. A recent study among Koreans reported that individuals expressing the HLA-B*44:03 allele may be highly susceptible to lamotrigine-induced SJS/TEN [111]. These alleles were suggested for use as screening markers to prevent cutaneous ADRs before the initiation of lamotrigine [110].

In the European population, a weak association was found between HLA-B*38 and lamotrigine-induced SJS/TEN (OR 80; *p* < 10^−6^) [112]. In other studies, several other alleles (i.e., HLA-B*5801, A*6801, Cw*0718, DQB1*0609 and DRB1*1301) were found to be weakly associated with lamotrigine-induced SCARs, but these results would need to be confirmed in a larger independent sample [113,114]. Another study failed to identify any single major HLA-related genetic risk factor for lamotrigine-induced SCARs in patients of European origin [113].

### 8.4. Phenobarbital-Induced SJS/TEN SCARs

Phenobarbital is one of the major causes of hypersensitivity to aromatic anticonvulsants in children. Patients with phenobarbital hypersensitivity may present with maculopapular rashes or SCARs, including SJS, TEN or DRESS. The incidence of aromatic anticonvulsant hypersensitivity is estimated to vary from 1/1000 to 1/10,000 [115], with a high mortality rate, especially in patients with SCARs [116]. Phenobarbital, the first-line anticonvulsant that is commonly used in seizure disorders in Thai children, is also one of the most common causes of SCARs in Thailand. Recently, there has been increasing evidence of the role of pharmacogenetics in predicting anticonvulsant-induced SCARs. However, there have only been a limited number of studies investigating the association between HLA genotypes and phenobarbital hypersensitivity. A small study from Japan demonstrated the association of HLA-B*51:01 and phenobarbital-induced SJS/TEN [117]. Eight patients who developed SJS/TEN reactions with the use of phenobarbital were recruited from the Japan Severe Adverse Reactions (JSAR) research group and RIKEN. One of them also had also received phenytoin. The onset of reaction after the initiation of the drug was 15.1 ± 7.1 days, which was slightly longer than those with reactions induced by phenytoin. Compared to healthy Japanese volunteers (n = 2878), six out of these eight patients carried HLA-B*51:01 and a significant association was observed between this allele and phenobarbital-induced SJS/TEN (OR 16.71, 95% CI 3.66–83.1) [117].

There is a potential cross-reactivity between use of aromatic AEDs in developing SCARs, possibly explained by the ‘hapten hypothesis’ mentioned earlier. Several studies have reported cases of patients with similar reactions following the use of two aromatic AEDs, while also presenting with the HLA-B*15:02 allele. A study by Locharernkul et al. [70] reported cases of MPE in three patients with the use of both CBZ and PHT. Meanwhile, Wang et al. found cross-reactivity towards aromatic AEDs in two Han Chinese patients who were both positive for HLA-B*15:02 [118]. Despite these reports, there is no definitive assumption on the magnitude and risk of AED-related cross-reactivity, partly because the use of another aromatic AED would have been avoided if the patients had a previous history of AED-induced SCARs.

## 9. Cytochrome P450 Enzymes and the Risk of SCAR

Cytochrome P450 is a superfamily of enzymes, including three main CYP families, CYP1, CYP2 and CYP3, which is responsible for the metabolism of a wide range of drugs [119,120]. Human beings have 57 CYP genes and 33 pseudogenes arranged into 18 families and 42 subfamilies. The P450 isoforms vary in abundance in the liver; however, CYP2C9, CYP2D6 and CYP3A4 account for 60–70% of all phase I biotransformations of drugs [121,122].

CYP2C9 and CYP2C19 are two of the most studied enzymes with clinically significant genetic variations (Table 3). The CYP2C9 gene is highly polymorphic, with at least 60 variants found in different populations [122]. CYP2C9*2 and *3 alleles have been associated with reduced enzymatic activity compared to the wild-type allele (CYP2C9*1) by 12% and 5% for *2 and *3, respectively [123]. Both alleles differ from the wild type by a single point mutation; CYP2C9*2 is characterized by a C416T SNP in exon three, resulting in an Arg144Cys amino acid substitution, whereas CYP2C9*3 expresses A1061C in exon 7, causing an Ile359Leu substitution [123].

Both of these alleles have proven to be determinants of significantly impaired metabolism of many CYP2C9 substrates, including phenytoin [124,125]. Phenotyping experiments showed that on a single dose of phenytoin, carriers build up approximately 30% higher serum levels compared to a person homozygous for the wild-type allele [126]. Since phenytoin has a narrow therapeutic index, this may have implications for the effect of the drug. A genome-wide-association study (GWAS) followed by direct sequencing of the CYP2C9 gene in patients from Taiwan, Malaysia and Japan showed that the missense variant CYP2C9*3 was associated with a 93–95% reduction in phenytoin clearance and that patients had phenytoin-induced SCARs. In addition to the CYP2C9 genotype, factors such as renal insufficiency, hepatic dysfunction and the concurrent use of substances that compete with or inhibit the enzymes contribute to variations in phenytoin clearance and/or to the risk of developing adverse effects, as delayed clearance of phenytoin was observed in the absence of CYP2C9C*3 [71].

In a study of 60 epileptic Dutch patients, the results demonstrated a strong association between the CYP2C9 allelic variables and the phenytoin dose requirements. The allele frequencies reported in this population were 76.7% for CYP2C9*1, 14.2% for CYP2C9*2 and 9.2% for CYP2C9*3, which were comparable to the frequencies reported in other studies of Caucasian populations [127]. Genotyping was suggested to be carried out at for any patients who might be prescribed phenytoin. Dosage adjustment based on the CYP2C9 genotype, especially prior to therapy, would be beneficial to lower the risk of concentration-dependent drug intoxication in carriers [127]. The defective CYP2C9 and/or CYP2C19 alleles could affect not only the pharmacokinetics but also the pharmacodynamics of phenytoin and CBZ therapy [128]. The FDA-approved drug label for phenytoin states that consideration should be given to avoiding phenytoin as an alternative for carbamazepine in individuals positive for HLA-B*15:02 because variant CYP2C9 alleles may contribute to unusually high levels of phenytoin [129]. For CYP2C19, three polymorphic alleles, CYP2C19*1 (wild type), CYP2C19*2 and CYP2C19*3, were identified to be relevant in the changes in drug metabolism. There were interethnic differences in CYP2C19 alleles observed in the Asian populations, particularly among the Chinese [9] and Japanese [130]. In a study conducted by Manuyakorn among Thai patients treated with CBZ and PHT, although the result was not statistically significant (OR 2.5, 95% CI 0.96–67.3; *p* = 0.06) [11], he showed that the patients with CYP2C19*2 variant had a higher likelihood of developing SCARs compared to patients with wild-type CYP2C19. It was also found that the CYP-catalysed metabolism of antiepileptics was increased in children [9,11].

**Table 3:** Cytochrome P450 allele frequency for various ethnicities.

AEDs	Population/Ethnicity	Genetic Variation	Allele Frequency	References
Carbamazepine	Thai	CYP2C19*2	0.29	[11]
	Korean	CYP3A5*3	0.237	[131,132]
	Japanese	*CYP3A5*	7	[128,133]
Phenytoin	Japanese	CYP2C9	0	[134]
	Caucasian	CYP2C9*2	0.15	[127]
	Caucasian	CYP2C9*3	0.07	[127]
	Indian	CYP2C19*2	4.5	[135,136]
		CYP2C19*3	10.1	
	Thai	CYP2C 19*2	0.27	[102,137]
	Thai	CYP2C19*3	0.02	[102,137]
	Malay	CYP2C9*1	0.9407	[134,138]
Phenobarbital	Japanese	*CYP2C19*	0.26	[139]
	Japanese	*CYP2C9*	0.966	[140]

## 10. Clinical Application of Pharmacogenetic Testing in the Prevention of SCARs

Genetic testing is able to predict a patient’s risk of developing ADRs, especially SCARs, and this knowledge could facilitate further strategies to avoid this complication. Pharmacogenetic testing is a type of genetic test consisting of a panel of genes important for the pharmacokinetics and pharmacodynamics of a drug, and is an attractive option for pre-emptive screening upon the initiation of a drug. This test can be used to identify individuals who are at risk of severe idiosyncratic adverse events, those who may not benefit from the therapy (e.g., non-responders) and the metabolism profile (e.g., fast versus slower metabolizers), which would ultimately allow for the individualization of drug dosage. This information would be useful in determining the choice of therapy and alternative strategies for treatment. Although there has been great interest in pharmacogenetics, there is a large gap in the knowledge on actionable variants (results which can change treatment), and the use of genetic testing in the clinical setting is limited to a few variants and drugs.

At present, several pharmacogenetic markers associated with drug hypersensitivity have been successfully identified and utilised in clinical practice. Growing evidence suggests that for patients with documented high genetic risks, pharmacogenomic testing is an efficient preventive tool. Currently, national health insurance organizations in Taiwan, Hong Kong, Singapore, Thailand and mainland China have approved pre-emptive genetic testing for the HLA-B*15:02 allele among new CBZ users [141,142]. Moreover, the U.S. FDA also suggests that HLA-B*15:02 testing be performed before patients are treated with oxcarbazepine. In addition to HLA-B*15:02, testing of the HLA-A*31:01 allele before the initiation of CBZ was also proposed among patients of Asian descent.

Screening for pharmacogenetic markers before the initiation of CBZ and PHT was shown to be cost-effective in addressing the risks of ADR. As examples, countries like Thailand, Singapore and Taiwan had performed pharmacogenetic screening for HLA alleles, and have shown significant reduction in the number of SJS/TEN cases reported with the use of risky drugs, not only limited to AEDs. As an example, HLA screening prior to the use of CBZ was implemented by the Singaporean Health Sciences Authority (HSA) in 2013 [141,142]. As a result of this national screening, a significant reduction of SJS/TEN cases was observed among patients on CBZ, from a baseline of ~18 cases per year (within 4 years) to only one case per year, just four years after the implementation of the HLA testing service [143,144]. In a Taiwanese study conducted on 4877 epilepsy patients prior to the initiation of CBZ, 7.7% of them were found to be positive for HLA-B*15:02. After 2 months of monitoring for symptoms of cutaneous reactions, a mild, transient rash was found to develop in 4.3% of the subjects, but SJS/TEN did not develop in any of the HLA-B*15:02-negative subjects receiving the same drug. This was a significant reduction from the historical incidence of 0.23% in one year [143].

**Figure 3:**
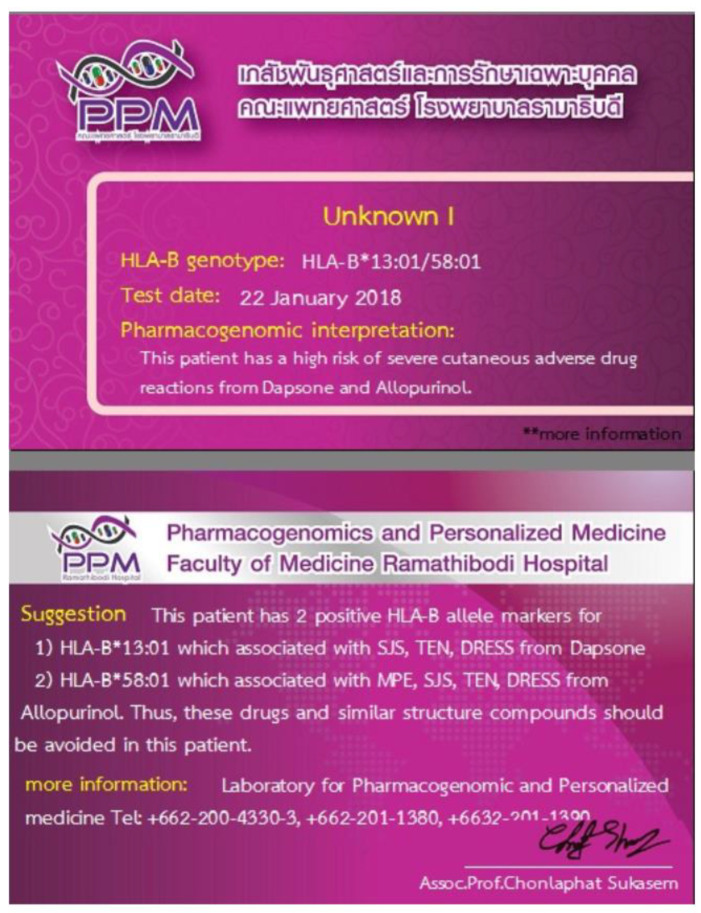
The pharmacogenetic card as part of pharmacogenetic implementation practice in Thailand. Patients’ pharmacogenetic information is entered into the pharmacogenetic card, a purple rectangle and wallet-sized card that they carry around and show to their future healthcare providers, such as physicians and pharmacists.

Meanwhile, in Thailand, pre-emptive genetic testing is also offered at Ramathibodi Hospital in Bangkok prior to the prescription of antiepileptics and other relevant drugs (Figure 3). The population frequency of HLA allele risk, predictive value, cost of genotyping and cost of alternative drugs were postulated to be the key factors influencing cost-effectiveness. Moreover, the availability and accessibility of these alternative drugs are crucial in maintaining the relevance of this pre-emptive testing and to safeguard patients.

Clinical Pharmacogenetics Implementation Consortium (CPIC) publishes genotype-based drug guidelines to help clinicians better understand how genetic test results could be used to optimize drug therapy. CPIC is focused on well-known examples of pharmacogenomic associations that have been implemented in selected clinical settings. Each CPIC guideline adheres to a standardized format and includes a standard system for grading evidence linking genotypes to phenotypes, and assigning a level of strength to each prescription recommendation. CPIC guidelines contain the necessary information to help clinicians translate patient-specific diplotypes for each gene into clinical phenotypes or drug dosage groups [144].

Several countries have implemented the pre-emptive pharmacogenetic screening of HLA allele for SCAR prevention, including Thailand, Singapore and Taiwan, as mentioned above. These countries have shown significant reductions in the number of SJS/TEN cases reported with the use of risky drugs, not only limited to AEDs. These implementation initiatives were shown to be supported by the authorities, and were incorporated as part of pharmacovigilance programs. Indeed, proactive steps should be taken to bridge pharmacogenetic knowledge and clinical application, particularly in the scope of preventable adverse drug reactions.

## 11. Conclusions

Cutaneous adverse drug reaction is a significant health burden, especially SCARs, which can be life threatening. Several immunological response mechanisms have been proposed to be involved in these reactions. Based on the current evidence, genetic screening is the best way forward as a preventive strategy against SCAR risks. An improved understanding of the mechanistic pathways of drug-induced SCARs and the pharmacokinetics of the offending drugs may provide insight into other potential markers in determining the risk of SCARs. While other possibly relevant genetic markers may be associated with AED-induced SCARs (e.g., variations in CYP2C19 genes), only a few HLA gene markers are currently included in pre-emptive genetic tests prior to the initiation of these drugs. In line with efforts towards precision medicine, continuous research in discovering genetic markers and performing cost–benefit analyses of pre-emptive screening may further facilitate preventive strategies in clinical practice.

## Figures and Tables

**Table 1:** Ethnicity and phenotype specific association between HLA allele and AED-induced SCARs.

AEDs	*HLA* Alleles	Population/Ethnicity	Phenotype	Reference
Carbamazepine	HLA-A*02:01	Iranian	SJS	[55]
	HLA-A*31:01		DRESS	
			MPE	[56,57,58,59,60]
		Caucasian	SJS/TEN	
		Japanese		
			DRESS,	[60]
			SJS/TEN	
		Korean		
			DRESS,	
			MPE,	
		Han Chinese		
	HLA-A*51:01	Iranian	SJS	[55]
	HLA-B*15:02	Han Chinese	SJS/TEN	[9,51,61,62,63,64]
		Thai		
		Malay		
		Indian		
		Vietnamese		
		Indonesian		
	*HLA-B* *∗* *15:08*	Indian	SJS/TEN	[65]
	HLA-B*15:11	Japanese	SJS/TEN	[66]
		Korean		[58]
	HLA-B*15:18	Japanese	SJS/TEN	[67]
	HLA-B*15:21	Filipino	SJS/TEN	[68]
Phenytoin	HLA-B*15:02	Han Chinese	SJS/TEN	[69]
		Thai		[70]
		Malay		[71]
	HLA-B*15:13	Malay	SJS/TEN	[71]
Lamotrigine	HLA-B*15:01	Han Chinese	SJS/TEN	[69]
				[72]
Phenobarbital	HLA-B*13:01	Thai	SJS/TEN	[73]
		Han Chinese		
	HLA--B*51:01	Japanese	SJS/TEN	[11]

Abbreviations. SJS: Stevens-Johnson syndrome; TEN: toxic epidermal necrolysis; DRESS: drug reaction with eosinophilia and systemic symptoms; MPE: maculopapular exanthema.

**Table 2:** HLA-A and HLA-B allele frequency for various ethnicities.

HLA Alleles	Population/Ethnicity	Allele Frequency	Reference
**HLA-A**			
HLA-A*02:01	Iranian	0.202	[74]
HLA-A*31:01	Caucasian	0.0214	[75]
	Japanese	0.084	[76]
	Korean	0.0562	[77]
	Han Chinese	0.0307	[78]
**HLA-B**			
HLA-B*13:01	Thai	0.210–0.0410	[79,80]
	Han Chinese	0.0405	[81]
HLA-B*15:01	Han Chinese	0.038	[82]
HLA-B*15:02	Han Chinese	0.0190–0.1240	[82,83]
	Thai	0.084	[84]
	Malay	0.1225	[85]
	Indian	0.013	[86]
	Vietnamese	0.135	[87]
	Indonesian	0.122	[88]
HLA-B*15:11	Japanese	0.0088	[76]
	Korean	0.0166	[77]
HLA-B*15:13	Malay	0.0599	[85]
HLA-B*15:18	Japanese	0.0152	[89]
HLA-B*15:21	Filipino	0.01	[90]
HLA-B*51:01	Japanese	0.07–0.9	[91]

## Data Availability

Not applicable. This review did not report any new data.
